# WELL-BEING AND ADVANCE DIRECTIVES AMONG PROLONGED MECHANICAL VENTILATION PATIENTS AT HOME VERSUS HOSPITAL

**DOI:** 10.1093/geroni/igz038.1679

**Published:** 2019-11-08

**Authors:** Jochanan Stessman, Esther-Lee Marcus, Jeremy M Jacobs

**Affiliations:** 1 Dept of Geriatrics and Rehabilitation, Jerusalem Institute for Aging Research, Hadassah Hebrew University Hospital, and Clalit Health services, Jerusalem, Israel; 2 Head, Chronic Ventilator-dependent Division, Herzog Hospital, Jerusalem, Israel

## Abstract

Despite increasing numbers of older patients requiring Prolonged Mechanical Ventilation (PMV), little is known concerning their mood, well-being, distressing symptoms and attitudes towards ventilation. Furthermore differences may exist according to place of care- whether Home Hospital or Hospital Long Term Care (HLTC). These issues were addressed using the revised Edmonton Symptom Assessment System (r-ESAS)(10 items, max severity score=100), and Short Geriatric Depression Scale, in a study of the majority of PMV patients (n=120/123) aged ≥18 (range 18-96 years) all Clalit Health Service insurees, in Jerusalem. Communicative patients were interviewed (40/46 at home, 22/74 in HLTC, average age 54 vs.73 years, p<0.01). The following symptoms (dichotomized to “not-a-problem”/“problematic”) were frequently reported as “not-a-problem” among patients (Home,HLTC): tiredness (59%,58%), poor appetite (95%,90%), pain (69%,84%), drowsiness (77%,90%), nausea (85%,84%), and shortness of breath (82%,90%). General well-being (categorized to good/mild/moderate/severe impairment) was reported more frequently as good or mildly impaired at Home vs. HLTC (54% vs.26%), as was lower frequency of depression (34.4% vs. 44.4%, p=0.049). The total r-ESAS score was similar irrespective of setting: Home-24.9/100 (inter quartile range {IQR} 13.5-32.5) vs. HLTC-23.7/100 (IQR 17.5-32),p=0.74. The majority (119/120) of patients were without advanced directives prior to initiation of PMV. When asked if they had to choose again, 82% and 91% of communicative patients at home vs. HLTC would opt again for ventilation, as would 75% of caregivers of uncommunicative patients. Our findings emphasize the resilience and low levels of distressing symptoms among PMV patients, and may contribute to the decision-making process concerning advanced directives.

